# *Staphylococcus aureus* Extracellular Adherence Protein Triggers TNFα Release, Promoting Attachment to Endothelial Cells via Protein A

**DOI:** 10.1371/journal.pone.0043046

**Published:** 2012-08-15

**Authors:** Andrew M. Edwards, Maria Gabriela Bowden, Eric L. Brown, Maisem Laabei, Ruth C. Massey

**Affiliations:** 1 Department of Biology and Biochemistry, University of Bath, Bath, United Kingdom; 2 Center for Infectious and Inflammatory Diseases, Texas A&M Health Science Center, Houston, Texas, United States of America; 3 Center for Infectious Disease, University of Texas School of Public Health, Houston, Texas, United States of America; University of Texas-Houston Medical School, United States of America

## Abstract

*Staphylococcus aureus* is a leading cause of bacteraemia, which frequently results in complications such as infective endocarditis, osteomyelitis and exit from the bloodstream to cause metastatic abscesses. Interaction with endothelial cells is critical to these complications and several bacterial proteins have been shown to be involved. The *S. aureus* extracellular adhesion protein (Eap) has many functions, it binds several host glyco-proteins and has both pro- and anti-inflammatory activity. Unfortunately its role *in vivo* has not been robustly tested to date, due to difficulties in complementing its activity in mutant strains. We previously found Eap to have pro-inflammatory activity, and here show that purified native Eap triggered TNFα release in whole human blood in a dose-dependent manner. This level of TNFα increased adhesion of *S. aureus* to endothelial cells 4-fold via a mechanism involving protein A on the bacterial surface and gC1qR/p33 on the endothelial cell surface. The contribution this and other Eap activities play in disease severity during bacteraemia was tested by constructing an isogenic set of strains in which the *eap* gene was inactivated and complemented by inserting an intact copy elsewhere on the bacterial chromosome. Using a murine bacteraemia model we found that Eap expressing strains cause a more severe infection, demonstrating its role in invasive disease.

## Introduction

*Staphylococcus aureus* is a commensal bacterium that asymptomatically colonises the nose either permanently or transiently of ∼60% of humans [1]. However, *S. aureus* is also responsible for a wide range of superficial and invasive infections that can result in significant morbidity and mortality [2]. The ability to cause such diverse afflictions is likely due to the huge number of different virulence factors that *S. aureus* expresses including toxins, adhesins, invasins and immune evasins [2], [3].

Whilst most *S. aureus* infections are superficial and self limiting, bacterial entry into the bloodstream via wound infections or colonisation of indwelling medical devices can lead to bacteraemia [4]. This is, in itself, a serious condition that can lead to sepsis with the release of high levels of pro-inflammatory cytokines [5], [6]. However, one of the hallmarks of *S. aureus* bacteraemia is the frequent development of secondary infections such as infective endocarditis, osteomyelitis and abscess formation in organs and tissues [7].

The process by which *S. aureus* leaves the bloodstream and seeds into remote sites is not fully understood and may occur via a number of very different mechanisms [8]. We recently demonstrated that the fibronectin-binding domain of fibronectin-binding protein A (FnBPA) is associated with endothelial cell invasion and bacterial penetration into the kidneys in a murine sepsis model [9]. However, whilst the vast majority of *S. aureus* isolates express functional FnBPs, the well characterised *S. aureus* strain Newman does not [10]. Although strain Newman contains both the *fnbA* and *fnbB* genes, each contains mutations that result in premature stop codons and truncation of FnBPA and FnBPB before the Sortase A recognition motif LPXTG [10]. As such, *S. aureus* Newman FnBPs are not anchored to the cell wall, and the bacterium does not bind fibronectin (Fn) [10]. It does, however, retain the ability to disseminate into the kidneys and form abscesses in murine bacteraemia models, suggesting that it possesses an alternative to FnBP-mediated dissemination [11], [12].

Despite the lack of FnBPs, recent work has reported that *S. aureus* Newman is able to attach to and invade endothelial cells. This occurs via the Extracellular adherence protein (Eap), which is encoded by the vast majority of *S. aureus* strains, but not other staphylococci [13], [14]. Eap consists of 4–6 repeats of 110 amino acids with high sequence identity but doesn’t encode the Sortase A recognition motif LPXTG [13]. Despite this, a proportion of secreted Eap attaches to the staphylococcal cell wall and mediates attachment to, and invasion of, host cells via a bridging mechanism between host and microbe [14]–[18]. Eap expression *in vivo* has been demonstrated by RNA and Western-blot analyses of *S. aureus* wound infections, as well as the detection of anti-Eap antibodies in patients [19]–[21]. Expression is strongly regulated by the two component signalling system SaeRS and to a lesser extent by Agr and SarA [22].

In addition to its role as an adhesin and invasin, Eap also has immuno-modulatory activity. Secreted Eap binds to ICAM-1, blocking the adhesion of blood monocytes and T-cells to activated endothelial cells [23]–[25]. Although this is inherently anti-inflammatory, the binding of Eap to ICAM-1 on the surface of peripheral blood mononucleocytes triggers the release of pro-inflammatory TNFα and IL-6 [26]. Despite structural homology to superantigens, Eap does not exhibit superantigen activity [27]–[29]. Although many studies have characterised the interaction of *S. aureus* with host cells, there is relatively little information on the role of the host immune response in modulating bacterial attachment and invasion and with Eap’s dual activity as an adhesin and a immuno-modulator we hypothesised that it may be important in this aspect of disease.

## Results

### Eap Triggers TNFα Release in Whole Human Blood

**Figure 1:**
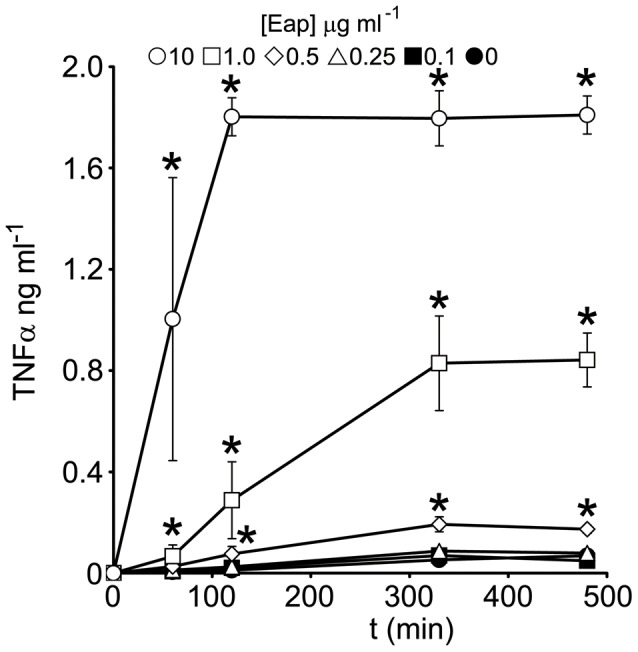
Eap triggers TNFα release in whole human blood in a dose-dependent manner. Whole human blood was incubated with native Eap at various concentrations (0–10 µg ml^−1^) for up to 480 min and TNFα production measured by ELISA. Values indicate the mean average of 3 independent experiments performed in duplicate. Error bars represent the standard deviation of the mean. Values that significantly different from blood incubated in the absence of Eap at identical time points are indicated (*).

We have previously shown that PBMCs secrete TNFα and IL-6 in response to purified native Eap [26]. However, whole blood is significantly more complex and we therefore sought to determine and quantify the effect Eap has on pro-inflammatory activity using whole human blood. Freshly drawn human blood was incubated with increasing concentrations of Eap. Concentrations up to 0.25 µg ml^−1^ failed to trigger significant release of TNFα (fig. 1). However, concentrations ≥0.5 µg ml^−1^ triggered a dose and time-dependent increase in TNFα secretion (fig. 1).

### TNFα Promotes *S. aureus* Adhesion to Endothelial Cells in a Dose-dependent Manner

**Figure 2:**
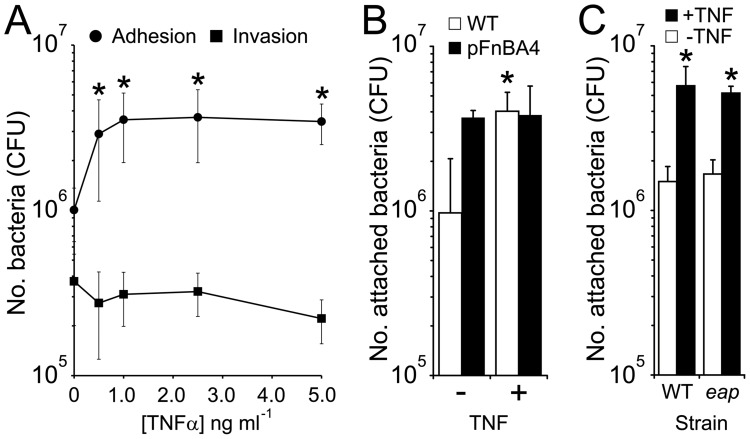
TNFα promotes *S. aureus* attachment to endothelial cells in a dose-dependent manner. The attachment to (circles) or invasion of (squares) endothelial cells by wild-type *S. aureus* Newman was examined after cells were pre-treated with various concentrations of TNFα (A). The attachment of *S. aureus* Newman WT to endothelial cells (pre-treated or untreated with TNFα) was compared with a WT strain expressing *fnbA* from a multicopy plasmid (pFnBA4) (B). *S. aureus* Newman wild-type and Δ*eap* mutant were compared for their ability to bind to endothelial cells (pre-treated or untreated with TNFα) (C). Values indicate the mean average of 3 independent experiments performed in duplicate. Error bars represent the standard deviation of the mean. Values that are significantly different from experiments where endothelial cells were incubated in the absence of TNFα are indicated (*).

Eap has been shown by several groups to facilitate invasion of human cells. To determine whether increased TNFα levels affects this, we pre-treated cultured endothelial cell monolayers with the cytokine prior to the addition of *S. aureus* strains and measured bacterial adhesion and invasion. In keeping with previous work [39], pre-treatment of endothelial cells with TNFα [1–5 ng ml^−1^] led to a dose-dependent increase in bacterial adhesion up to 4-fold at the highest concentration (fig. 2a). As described above, Eap (1–10 µg ml^−1^) triggered TNFα release that reached concentrations of approximately 1–2 ng ml^−1^ (fig. 1). Pre-treatment of endothelial cells with similar concentrations lead to enhanced attachment that was ∼3.5-fold greater than untreated cells (fig. 2a). By contrast, TNFα pre-treatment of endothelial cells did not lead to higher levels of bacterial internalisation at any of the concentrations examined (fig. 2a).

Previous work has implicated FnBPA in *S. aureus* attachment to the endothelium [30], [31]. To examine whether this TNFα induced, increased attachment was equivalent to that mediated by FnBPA we expressed a functional *fnbA* gene in Newman and compared adhesion with and without TNFα. Newman expressing FnBPA bound endothelial cells at significantly higher levels than wild-type (WT) Newman alone (fig. 2b). However, this difference was abrogated when examining adhesion to TNFα- pre-treated cells; WT Newman cells bound equally well to pre-treated cells as WT FnBPA+ *S. aureus* (fig. 2b). Furthermore, the enhanced adhesion was not mediated by Eap; the *eap* mutant bound treated and untreated endothelial cells at similar levels to WT *S. aureus* Newman (fig. 2c). This demonstrates that Newman is able to bind efficiently to endothelial cells in the absence of FnBPA and Eap following exposure of endothelial cells to Eap induced levels of TNFα.

### TNFα-enhanced Adhesion Occurs via a Mechanism that Involves Staphylococcal Protein A and Endothelial gC1qR/p33

**Figure 3:**
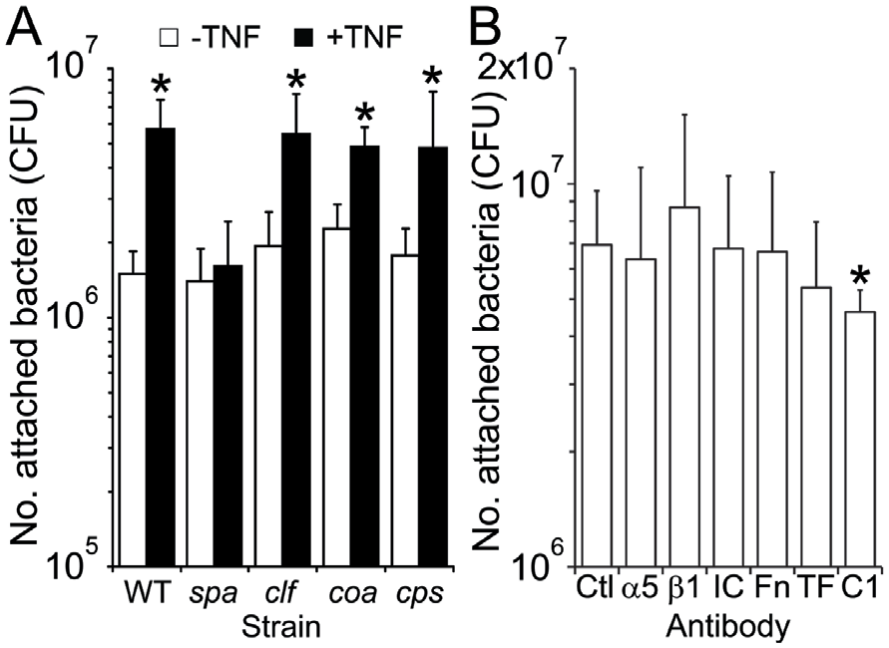
Protein A mediates increased attachment to endothelial cells via a mechanism involving gC1qR/p33. A selection of strains deficient in cell-surface proteins Protein A (spa), Clumping factor A (*clf*), Coagulase (*coa*)) or capsular polysaccharide (*cps*) were assessed for their ability to bind to either TNFα pre-treated or untreated endothelial cells (A). Attachment of *S. aureus* Newman to TNFΑ-treated cells in the absence (Ctl) or presence of various antibodies, including α5 (α5) and β1 (β1) integrin subunits, ICAM-1 (IC), Fibronectin (Fn), Tissue factor (TF) and gC1qR/p33 (C1) was also determined (B). Values indicate the mean average of 3 independent experiments performed in duplicate. Error bars represent the standard deviation of the mean. Values that are significantly different (p<0.05) from experiments where endothelial cells were incubated in the absence of TNFα (A) or antibodies (B) are indicated (*).

To identify the *S. aureus* adhesin responsible for mediating the enhanced adhesion to TNFα-pretreated cells we examined the attachment of *S. aureus* Newman strains deficient in a variety of surface proteins (Eap, Spa, ClfA, Coa) or capsule (CPS) (fig. 3). All strains bound equally well to untreated endothelial cells (fig. 3). However, the only strain that did not exhibit enhanced adhesion to TNFα-treated endothelial cells was the protein A mutant (*spa*) (fig. 3).

TNFα has been reported to up-regulate expression of a number of different receptors on the surface of endothelial cells, including gC1qR/p33, which has previously been reported to be a receptor for protein A [32]. To identify the molecule on the endothelial cell surface to which protein A was binding we treated endothelial cells with TNFα followed by antibodies against ICAM-1, Tissue factor (TF), Integrin subunits α5 and β1, Fibronectin (Fn) and gC1qR/p33 (C1) and subsequently assessed the ability of Newman to bind the cells. Only antibodies against gC1qR/p33 displayed any significant inhibitory activity (18% inhibition, p = 0.04).

### Complementation of the *eap* Mutation and Characterisation of Strains

**Figure 4:**
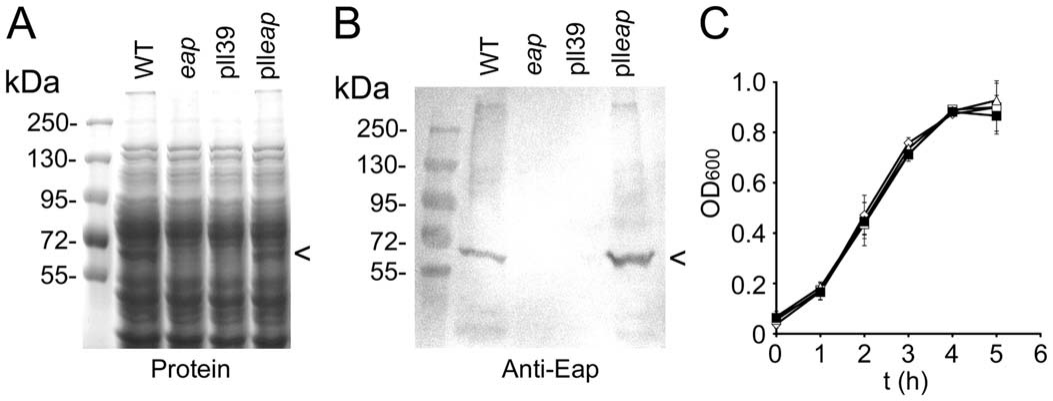
Complementation of *eap* deletion. *S. aureus* strain Newman wild-type (WT), mAH12 (*eap*), mAH12pll39 (pll39) and mAH12pll*eap* (pll*eap*) were assessed for Eap production by SDS-PAGE (A) and Western blotting using anti-Eap antibodies (B). The position of bands corresponding to Eap is indicated (<). The growth rates of the four strains were compared to ensure that complementation did not have an adverse effect (C).

A *S. aureus* Newman *eap* isogenic deletion mutant (mAH12) has been created and reported previously [16]. However, complementation of the mutation, by expression of *eap* on a multicopy plasmid has produced mixed results, often resulting in low levels of Eap expression as well as plasmid instability [16], [33]. To overcome this we introduced a single copy of *eap* into the *geh* gene on the chromosome of *S. aureus* Newman using a lysogenic bacteriophage L54a site-specific recombination system [34]. Plasmid pll39 containing the *eap* gene (pll*eap*) was inserted into the L54a attB insertion site within the *geh* gene. An empty copy of pll39 inserted into the same location was also produced to act as a negative control. SDS-PAGE and Western-immunoblot analysis using polyclonal anti-Eap antisera demonstrated that *S. aureus* Newman wild-type and complemented strain mAH12(pll*eap*) both expressed Eap and at similar levels (fig. 4a and b). By contrast, the *eap* mutant mAH12 and the complementation control strain mAH12(pll39) did not express Eap. There were no apparent differences in expression of other cell wall-associated proteins as a result of the *eap* complementation (fig. 4a). The insertion of pll*eap* onto the chromosome of *S. aureus* appeared to be stable – after 10 consecutive sub-cultures of stationary-phase broth cultures in the absence of antibiotics the number of tetracycline resistant colony forming units (CFU) was identical to the total number of CFU within the broth. Further analysis revealed that 99% of colonies on the antibiotic free plate were tetracycline resistant, demonstrating the stability of the construct (data not shown). Furthermore, the integration of pll39 and pll*eap* into the *geh* gene had no effect on bacterial growth rate (fig. 4c) or production of cytolytic toxins as determined by a T-cell lysis assay (data not shown, [35]).

### Eap Enhances the Severity of *S. aureus* Bacteraemia

**Figure 5:**
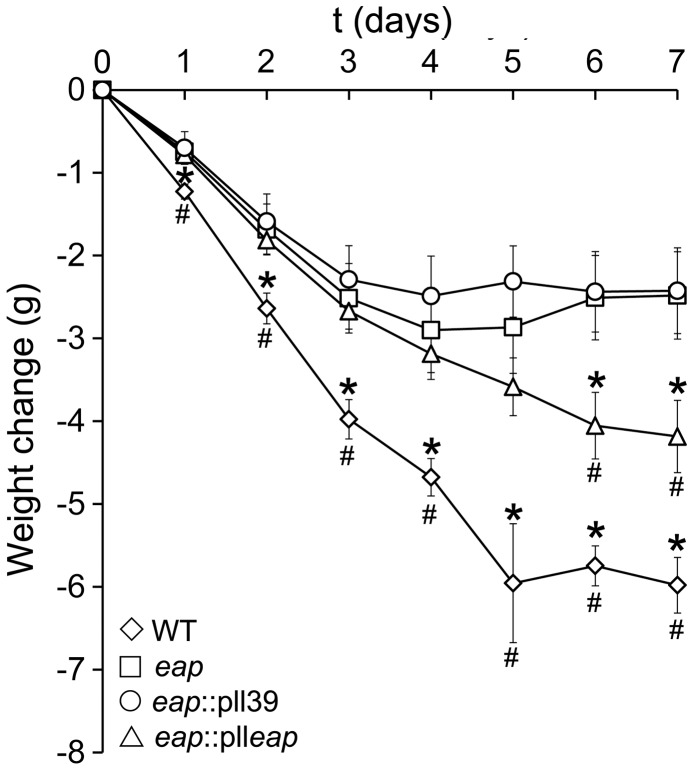
Eap is a virulence factor in a murine bacteremia model. Mice were challenged with Newman wild-type (WT), mAH12 (*eap-*), mAH12 (pll39) and mAH12 (pll*eap*) and their weight monitored over 7 days. Error bars represent the standard deviation of the mean. Values that are significantly different (p<0.05) from mAH12 (*eap-*) are highlighted with an *, and those significantly different (p<0.05) from mAH12 (pll39) with a #.

We hypothesised that the induction of TNFα during bacteraemia and subsequent enhancement of *S. aureus* attachment to the endothelium is likely to enhance disease severity. To test this we employed a murine sepsis model and assessed disease severity by monitoring weight loss over time. Previous studies using this model have shown a clear correlation between this marker and disease severity [9]. As expected, infection of mice with WT Newman resulted in a rapid weight loss that did not recover during the course of the assay and was significantly greater than that seen with the *eap* mutant at all time points (fig. 5). By contrast, infection with the *eap* mutant resulted in relatively modest weight loss that stabilised during the course of the assay (fig. 5, WT weight loss was significantly lower than *eap*, p<0.001 at day 7). As expected, the *eap* mutant strain containing the empty pll39 vector showed an almost identical weight loss profile to the *eap* mutant (fig. 5). However, complementation of the *eap* mutation with a copy of *eap* (pll*eap*) resulted in similar, although less pronounced, amount of weight loss to that caused by WT Newman, demonstrating the successful complementation of the mutation and verifying the role Eap plays in this aspect of disease (fig. 5).

## Discussion

*S. aureus* bacteraemia and the subsequent development of metastatic *S. aureus* infections involves a number of different processes including immune evasion, adhesion, escape from the bloodstream, multiplication and abscess formation [2], [3]. Each of these processes involves several different host and bacterial factors. A previous study has indicated a role for Eap in the later stages of abscess formation following bacteraemia [12]. In this work we demonstrate that Eap is also important during the early stages of bacteraemia and present evidence of a novel role for Eap in attachment to the endothelium.

Eap is a multifunctional protein with immune-modulatory, adhesive and invasive properties. We previously reported its role in inducing the expression of pro-inflammatory cytokines, and here we quantified this and examined the effect this might have on bacterial-host interactions. In keeping with previous studies using isolated PBMCs, Eap strongly triggered induction of TNFα in whole blood. Previous work showed that Eap interactions with ICAM-1 was responsible for triggering TNFα release. However, the interaction of Eap with ICAM-1 also has anti-inflammatory activity via its ability to block leukocyte and T cell extravasation. Indeed, Eap has been proposed as a potential therapeutic agent in the treatment of autoimmune inflammatory disorders such as multiple sclerosis and psoriasis [24], [35]. However, the ability of Eap to induce significant TNFα release suggests caution should be applied when assessing its utility in patient therapy.

Eap is not the only *S. aureus* trigger of TNFα release. Cell wall components including peptidoglycan and lipoteichoic acid stimulate immune cells to produce TNFα, as do the secreted phenol-soluble modulins and delta toxin [36]–[38]. However, by contrast to the cytolysins, which are expressed in stationary phase, Eap is secreted during exponential-phase growth [21]. As such, the contribution of Eap to stimulation of TNFα production very likely occurs during a different phase of the infection to cytolytic toxins.

The release of TNFαhas clear implications for the immune response to *S. aureus,* but it also has effects on the endothelial cells lining the blood vessels [39]. Attachment to the endothelium is a precursor to bacterial escape from the bloodstream and penetration into surrounding tissues [8]. Several studies have indicated a role for Eap in attachment to and invasion of host cells but the effect of inflammatory cytokines were unclear [15]–[18], [39]. Our data shows enhanced *S. aureus* attachment to endothelial cells when pre-treated with levels of TNFα found to be induced by the exposure of whole blood to Eap, which is in keeping with other work [40], [41]. Surprisingly, despite previous work showing that Eap mediates *S. aureus* attachment to, and invasion of, endothelial cells [16], [17] it did not appear to mediate adhesion to either unstimulated or stimulated endothelial cells in our assays. It is possible that this is due to significant differences between the cell line used in this study and those reported previously.

In this study we identified the *S. aureus* surface protein responsible for the enhanced binding to TNFα pre-treated cells as protein A, and the molecule on the endothelial cell surface as gC1qR/p33. Whilst protein A is best known as an immunoglobulin-binding immune evasin, it also has the ability to bind other human proteins such as Von Willebrand Factor [41]. Previous work has also shown that protein A binds to the surface of platelets [32]. The data reported here is consistent with other studies where anti- gC1qR/p33 monoclonal antibodies have been shown *in vivo* to block *S. aureus* adhesion to endothelial cells treated with TNFα in a murine model, and to reduce *S. aureus* colonization of the aorta in a rat model of infective endocarditis [42], [43].

Previous work [46] has identified gC1qR/p32 as a receptor for *Listeria monocytogenes* invasin InlB, indicating that this protein can support bacterial internalization. However, despite enhanced attachment of *S. aureus* to TNFα-pretreated endothelial cells, there was no significant increase in bacterial internalisation. There are three possible explanations for this. Firstly, the endothelial cell surface receptor mediating enhanced *S. aureus* adhesion is not capable of mediating bacterial internalisation. Secondly, the mechanisms responsible for *S. aureus* Newman invasion were not enhanced by TNFα pre-treatment and so were unable to internalise the extra bacteria attached to the cell. Thirdly, TNFα pre-treatment does increase invasion but also increases intracellular killing, resulting in no net difference in the number of viable intracellular bacteria. In keeping with this final possibility, previous work has shown that TNFα promotes *S. aureus* invasion of bovine endothelial cells via its effect on NF-KB but also promotes intracellular killing [44], [45].

Our data suggest that Eap may play a role in disease severity during the early stages of bacteraemia when the bacteria first interact with the endothelium. Previous attempts to conclusively demonstrate the activity of Eap *in vivo* have failed due to an inability to restore the phenotype by complementation, which is important in bacteria due to the polar effects that can occur when constructing mutants, and how readily secondary mutations can occur elsewhere on the chromosome. In an attempt to address this problem we constructed a strain in which the mutation was complemented by inserting an intact copy of the *eap* gene in the *geh* gene on the chromosome, which is more stable than using a plasmid. To examine the role of Eap early in bacteraemia we used an *in vivo* murine bacteraemia model. We show that WT Newman causes a more severe infection than the isogenic *eap* mutant strain mAH12 during the first few days of infection. For the first time, we were able to reliably test the virulence of a complemented strain in *in vivo* assays by creating a stable, chromosomal *eap* complemented strain in the *eap* mutant strain mAH12 background. Virulence was restored in the complemented strain, confirming the role of Eap during the early stages of bacteraemia.

In summary, our data demonstrate that Eap is important for pathogenesis during the early stages of bacteraemia and identify a novel mechanism by which Eap might enhance attachment to the endothelium; Eap-triggered TNFα release in the bloodstream paves the way for systemic *S. aureus* infection by up-regulation of host receptor gC1qR/p33 on the surface of endothelial cells, which is targeted by protein A.

## Methods

### Ethics Statement

Approval for experiments using human blood was granted by the Bath Research Ethics Committee (NHS National Research Ethics Services, reference 08/H0101/18). Donors gave informed consent in writing prior to the commencement of any procedures.

All animal experiments conformed to the National Institute of Health guidelines and were approved by the Institutional Animal Care Use Committee at the Texas A&M HSC Institute of Biosciences and Technology. All surviving mice were euthanized at day 7 post-inoculation. Criteria for determining morbidity/sickness in mice included hunched posture, decreased activity, ruffled fur and laboured breathing.

### Bacterial Strains and Culture Conditions

A list of the bacterial strains used can be found in table 1. *S. aureus* was cultured in Brain-Heart Infusion broth (Sigma) for 16 h at 37°C in air with shaking (180 rpm). Bacteria were pelleted by centrifugation, washed by resuspension in PBS and centrifuged again before a final resuspension in PBS to OD_600_ = 1.0. Bacterial CFU were enumerated on Tryptic-Soy agar plates. *E. coli* was grown in LB broth or on LB agar. Where necessary, tetracycline (3 µg ml^−1^ *S. aureus*/10 µg ml^−1^ *E. coli*) was included in the medium.

**Table 1:** Strains used in this study.

Strain	Relevant characteristics	Reference
***E. coli***		
DH5α	Host strain for pll39 undergoing genetic manipulation.	
Pll39	Plasmid allowing for single copy integration of DNA onto the *S. aureus* chromosome.	[34]
pll*eap*	Pll39 containing the *eap* gene and flanking DNA including promoter region.	This study
***S. aureus***		
Phage Φ11	Used for transduction of DNA between *S. aureus* strains.	[48]
pFnBA4	Plasmid containing the entire *fnbA* gene and promoter.	[49]
RN4220	Restriction deficient sub-cloning host strain. Tolerates exogenous DNA.	[50]
Newman WT	Wild type strain. Expresses Eap.	[51]
Newman mAH12	Newman Δ*eap* mutant strain (*eap*::*erm*B).	[16]
Newman mAH12(pll39)	Δ*eap geh*::pll39. *eap* deficient mutant strain with empty pll39 plasmid inserted into the L54a attBinsertion site within the *geh* gene.	This study
Newman mAH12(plleap)	Δ*eap geh*::plleap. *eap* deficient mutant strain with pll39 plasmid containing the *eap* gene inserted into theL54a attB insertion site within the *geh* gene. Expresses Eap.	This study
Newman WT pFnBA4	Newman wild-type strain expressing FnBPA.	This study
DU5873	Newman Δs*pa* mutant strain (*eap*::Tc^r^).	[52]
DU5852	Newman Δ*clfA* mutant strain (*clfA*::Tn*917* Em^r^).	[53]
DU5855	Newman Δ*coa* mutant strain (*coa*::Tc^r^).	[54]
DU5917	Newman Δ*cps* mutant strain (*eap*::Tn*917* Em^r^).	[55]

### Introduction of *eap* onto the Chromosome of *S. aureus* Newman Δ*eap* (mAH12)

The *eap* gene and flanking DNA including promoter and RBS was amplified from wild-type *S. aureus* Newman (NC_009641) using primers EapF (5′-TGGAGGATCCTGTTTTTTGAGTATAAAGATGCTG-3′) and EapR (5′-TGGACTGCAGATTATAGAACACAAATTCATTTGAAA-3′) designed to include BamH1 and Pst1 restriction sites respectively (underlined). These were used to ligate the amplified DNA into plasmid pll39 [33] to create pll*eap*. Ligation reactions were transformed into CaCl_2_ competent *E. coli* DH5α and transformants selected for on LB agar containing tetracycline. Plasmid pll*eap* was recovered using a Qiagen miniprep kit, checked by DNA sequencing, and transformed into *S. aureus* RN4220 by electroporation. Tetracycline resistant colonies were examined for integration of plleap (or pll39 only, which was employed as a control). Because pll39 can integrate into 1 of 2 sites, its location was determined using PCR as described previously [34]. A matched isolate containing pll39 integrated at the same site as a selected pll*eap* containing isolate was selected as a control strain. The integrated pll39 and pll*eap* were transduced into *S. aureus* strain Newman using phage 11 as described previously [47], [48]. Transductants were examined for pll39 or pll*eap* by PCR and phenotypic characterisation was performed as described below.

### Analysis of *S. aureus* Surface Proteins

*S. aureus* surface proteins were analysed as described previously [8]. Protein concentrations of supernatants were determined using the Bradford assay and 20 µg subjected to SDS-PAGE analysis on a 10% acrylamide gel, which was subsequently stained using EZblue (Sigma). Separated proteins were also blotted onto nitrocellulose membrane, which was subsequently blocked with 3% BSA. Eap was detected using polyclonal anti-Eap antibodies, followed by HRP-conjugated protein G and colourimetric detection with opti4CN (Biorad).

### T-cell Cytotoxicity Assay

The production of secreted cytotoxins by *S. aureus* strains was assessed using a T-cell cytotoxicity assay as described previously [35].

### Stability of Inserted DNA

To determine the stability of the inserted pll*eap* DNA, *S. aureus* Newman was sub-cultured 10 times in BHI broth without antibiotics and the number of tetracycline resistant CFU determined. In addition, sub-cultured *S. aureus* was plated onto antibiotic-free TSA plates and 100 colonies picked and re-plated onto TSA plates containing tetracycline to determine the % of resistant CFU.

### Purification of Eap

Native Eap was purified as described previously using a protocol that produces endotoxin-free protein [15], [26]. Eap preparations were concentrated and de-salted using centricon filters (Millipore) with a molecular weight cut-off of 10 kDa. Previous work has shown that this purification protocol does not liberate any other pro-inflammatory mediators from the surface of *S. aureus* [26]. The purity and identity of Eap was confirmed by SDS-PAGE and Western blot analysis as described above.

### Effect of Eap on TNFα Production in Whole Human Blood

Quantification of TNFα expression in whole human blood was performed as described previously [9]. Whole human blood (1 ml aliquots) was incubated with purified native Eap at indicated concentrations. At various time points, the blood samples were centrifuged to pellet cells and plasma recovered. TNFα concentration in samples was determined using a BD OptEIA ELISA kit (BD Biosciences, San Diego, USA) according to manufacturer’s instructions.

### Endothelial Cell Adhesion and Invasion Assays

Bacterial adhesion to and invasion of EA. hy926 endothelial cells was performed essentially as described previously with some modifications [9].

Endothelial cells were cultured in Dulbecco’s modified Eagles’ medium supplemented with foetal bovine serum (FBS, 10%) and l-glutamine (2 mM) in T75 flasks at 37°C and 5% CO2. Cells were liberated from flasks using trypsin-EDTA solution (Sigma), resuspended in culture medium and aliquoted into wells of a 24-well plate containing thermanox coverslips. Once the endothelial cell monolayer was >95% confluent (as determined by microscopy) coverslips were transferred to fresh wells containing DMEM supplemented with 10% FBS. Some wells also contained recombinant human TNF (0.5–10 ng ml^−1^). Cells were incubated for a further 4 hr at 37°C. Coverslips with attached endothelial cell monolayers were then dip-washed 3 times in DMEM and added to fresh wells containing DMEM 10% FBS and 10^8^ *S. aureus*. Bacteria were incubated with the cells for 120 minutes. Coverslips that were used to determine the total number of associated CFU (adherent and internalised) were dip washed 3 times and added to fresh wells containing 250 µl trypsin-EDTA solution for 10 min at 37°C in 5% CO2 to disrupt monolayers and break up bacterial aggregates. Cells were subsequently lysed by the addition of 250 µl Triton X-100 solution. Bacterial CFU were enumerated by serial dilution of endothelial cell lysates and plating onto TSA plates. Coverslips that were used to determine the number of internalised bacteria were incubated in 500 µl DMEM containing 10% FBS, Gentamicin (200 µg ml^−1^) and lysostaphin (5 µg ml^−1^) for 1 hr at 37°C in 5% CO_2_ to kill external bacteria. Coverslips were dip-washed 3 times, incubated in trypsin-EDTA, lysed with Triton X-100 and CFU enumerated as described above.

### Murine Bacteraemia Assay

Overnight cultures (200 µl) were used to inoculate CCY broth (10 ml in 50 ml flask), which were grown to OD_600_ = 0.8 (37°C, 225 rpm) before harvesting of bacteria by centrifugation. Bacteria were washed, resuspended in sterile PBS to the desired infection dose and the number of bacteria present in the inoculum verified by culturing serial dilutions on TSA plates. Mice were injected intravenously with approximately 10^7^ *S. aureus* in a volume of 500 µl. Mice were weighed daily for 7 days and weight change differences were analyzed for statistical significance using the Student’s *t*-test. All surviving mice were euthanized at day 7 post-inoculation. Criteria for determining morbidity/sickness in mice included hunched posture, decreased activity, ruffled fur and laboured breathing. All animal experiments conformed to the National Institute of Health guidelines and were approved by the Institutional Animal Care Use Committee at the Texas A&M HSC Institute of Biosciences and Technology.

## References

[1] Kluytmans J, van Belkum A, Verbrugh H (1997) Nasal carriage of *Staphylococcus aureus*: epidemiology, underlying mechanisms, and associated risks. Clin Microbiol Rev 10: 505–20. 9227864 10.1128/cmr.10.3.505 PMC172932

[2] Lowy FD (1998) *Staphylococcus aureus* infections. N Engl J Med 339: 520–532. 9709046 10.1056/NEJM199808203390806

[3] Gordon RJ, Lowy FD (2008) Pathogenesis of methicillin-resistant *Staphylococcus aureus* infection. Clin Infect Dis. 46: S350–9. 10.1086/533591 PMC2474459 18462090

[4] Carnicer-Pont D, Bailey KA, Mason BW, Walker AM, Evans MR, et al (2006) Risk factors for hospital-acquired methicillin-resistant *Staphylococcus aureus* bacteraemia: a case-control study. Epidemiol Infect 134: 1167–73. 16623990 10.1017/S0950268806006327 PMC2870517

[5] Bone RC (1994) Gram-positive organisms and sepsis. Arch Intern Med 154: 26–34. 8267486

[6] Fournier B, Philpott DJ (2005) Recognition of *Staphylococcus aureus* by the innate immune system. Clin Microbiol Rev 18: 521–40. 16020688 10.1128/CMR.18.3.521-540.2005 PMC1195972

[7] Fowler VG Jr, Olsen MK, Corey GR, Woods CW, Cabell CH, et al (2003) Clinical identifiers of complicated *Staphylococcus aureus* bacteremia. Arch Intern Med. 163: 2066–72. 10.1001/archinte.163.17.2066 14504120

[8] Edwards AM, Massey RC (2011) How does *Staphylococcus aureus* escape the bloodstream? Trends Microbiol 19: 184–90. 21227700 10.1016/j.tim.2010.12.005

[9] Edwards AM, Potts JR, Josefsson E, Massey RC (2010) *Staphylococcus aureus* host cell invasion and virulence in sepsis is facilitated by the multiple repeats within FnBPA. PLoS Pathog. 6: e1000964. 10.1371/journal.ppat.1000964 PMC2891841 20585570

[10] Grundmeier M, Hussain M, Becker P, Heilmann C, Peters G, et al (2004) Truncation of fibronectin-binding proteins in *Staphylococcus aureus* strain Newman leads to deficient adherence and host cell invasion due to loss of the cell wall anchor function. Infect Immun 72: 7155–63. 15557640 10.1128/IAI.72.12.7155-7163.2004 PMC529102

[11] Thammavongsa V, Kern JW, Missiakas DM, Schneewind O (2009) *Staphylococcus aureus* synthesizes adenosine to escape host immune responses. J Exp Med 206: 2417–27. 19808256 10.1084/jem.20090097 PMC2768845

[12] Cheng AG, Kim HK, Burts ML, Krausz T, Schneewind O, et al (2009) Genetic requirements for *Staphylococcus aureus* abscess formation and persistence in host tissues. FASEB J 23: 3393–404. 19525403 10.1096/fj.09-135467 PMC2747682

[13] Hussain M, Becker K, von Eiff C, Peters G, Herrmann M (2001) Analogs of Eap protein are conserved and prevalent in clinical *Staphylococcus aureus* isolates. Clin Diagn Lab Immunol 8: 1271–6. 11687475 10.1128/CDLI.8.6.1271-1276.2001 PMC96261

[14] Hussain M, von Eiff C, Sinha B, Joost I, Herrmann M, et al (2008) Eap Gene as novel target for specific identification of *Staphylococcus aureus* . J Clin Microbiol 46: 470–6. 18094124 10.1128/JCM.01425-07 PMC2238111

[15] Palma M, Haggar A, Flock JI (1999) Adherence of *Staphylococcus aureus* is enhanced by an endogenous secreted protein with broad binding activity. J Bacteriol 181: 2840–5. 10217776 10.1128/jb.181.9.2840-2845.1999 PMC93727

[16] Hussain M, Haggar A, Heilmann C, Peters G, Flock JI, et al (2002) Insertional inactivation of Eap in *Staphylococcus aureus* strain Newman confers reduced staphylococcal binding to fibroblasts. Infect Immun 70: 2933–40. 12010982 10.1128/IAI.70.6.2933-2940.2002 PMC128007

[17] Hussain M, Haggar A, Peters G, Chhatwal GS, Herrmann M, et al (2008) More than one tandem repeat domain of the extracellular adherence protein of *Staphylococcus aureus* is required for aggregation, adherence, and host cell invasion but not for leukocyte activation. Infect Immun 76: 5615–23. 18794290 10.1128/IAI.00480-08 PMC2583574

[18] Haggar A, Hussain M, Lönnies H, Herrmann M, Norrby-Teglund A, et al (2003) Extracellular adherence protein from *Staphylococcus aureus* enhances internalization into eukaryotic cells. Infect Immun 71: 2310–7. 12704099 10.1128/IAI.71.5.2310-2317.2003 PMC153217

[19] Athanasopoulos AN, Economopoulou M, Orlova VV, Sobke A, Schneider D, et al (2006) The extracellular adherence protein (Eap) of *Staphylococcus aureus* inhibits wound healing by interfering with host defense and repair mechanisms. Blood 107: 2720–7. 16317095 10.1182/blood-2005-08-3140 PMC1895382

[20] Joost I, Blass D, Burian M, Goerke C, Wolz C, et al (2009) Transcription analysis of the extracellular adherence protein from *Staphylococcus aureus* in authentic human infection and in vitro. J Infect Dis 199: 1471–8. 19351261 10.1086/598484

[21] Joost I, Jacob S, Utermöhlen O, Schubert U, Patti JM, et al (2011) Antibody response to the extracellular adherence protein (Eap) of *Staphylococcus aureus* in healthy and infected individuals. FEMS Immunol Med Microbiol 62: 23–31. 21251089 10.1111/j.1574-695X.2011.00783.x

[22] Harraghy N, Kormanec J, Wolz C, Homerova D, Goerke C, et al (2005) Sae is essential for expression of the staphylococcal adhesins Eap and Emp. Microbiology 151: 1789–800. 15941988 10.1099/mic.0.27902-0

[23] Chavakis T, Hussain M, Kanse SM, Peters G, Bretzel RG, et al (2002) *Staphylococcus aureus* extracellular adherence protein serves as anti-inflammatory factor by inhibiting the recruitment of host leukocytes. Nat Med 8: 687–93. 12091905 10.1038/nm728

[24] Haggar A, Ehrnfelt C, Holgersson J, Flock JI (2004) The extracellular adherence protein from *Staphylococcus aureus* inhibits neutrophil binding to endothelial cells. Infect Immun 72: 6164–7. 15385525 10.1128/IAI.72.10.6164-6167.2004 PMC517550

[25] Xie C, Alcaide P, Geisbrecht BV, Schneider D, Herrmann M, et al (2006) Suppression of experimental autoimmune encephalomyelitis by extracellular adherence protein of *Staphylococcus aureus* . J Exp Med 203: 985–94. 16585266 10.1084/jem.20051681 PMC2118278

[26] Scriba TJ, Sierro S, Brown EL, Phillips RE, Sewell AK, et al (2008) The *Staphyloccous aureus* Eap protein activates expression of proinflammatory cytokines. Infect Immun. 76: 2164–8. 10.1128/IAI.01699-07 PMC2346708 18332207

[27] Geisbrecht BV, Hamaoka BY, Perman B, Zemla A, Leahy DJ (2005) The crystal structures of EAP domains from *Staphylococcus aureus* reveal an unexpected homology to bacterial superantigens. J Biol Chem 280: 17243–50. 15691839 10.1074/jbc.M412311200

[28] Massey RC, Scriba TJ, Brown EL, Phillips RE, Sewell AK (2007) Use of peptide-major histocompatibility complex tetramer technology to study interactions between *Staphylococcus aureus* proteins and human cells. Infect Immun 75: 5711–5. 17938227 10.1128/IAI.00875-07 PMC2168368

[29] Haggar A, Flock JI, Norrby-Teglund A (2010) Extracellular adherence protein (Eap) from *Staphylococcus aureus* does not function as a superantigen. Clin Microbiol Infect 16: 1155–8. 19769600 10.1111/j.1469-0691.2009.03058.x

[30] Peacock SJ, Foster TJ, Cameron BJ, Berendt AR (1999) Bacterial fibronectin-binding proteins and endothelial cell surface fibronectin mediate adherence of *Staphylococcus aureus* to resting human endothelial cells. Microbiol 145: 3477–86. 10.1099/00221287-145-12-3477 10627045

[31] Kerdudou S, Laschke MW, Sinha B, Preissner KT, Menger MD, et al (2006) Fibronectin binding proteins contribute to the adherence of *Staphylococcus aureus* to intact endothelium in vivo. Thromb Haemost 96: 183–9. 16894462

[32] Nguyen T, Ghebrehiwet B, Peerschke EI (2000) *Staphylococcus* aureus protein A recognizes platelet gC1qR/p33: a novel mechanism for staphylococcal interactions with platelets. Infect Immun 68: 2061–8. 10722602 10.1128/iai.68.4.2061-2068.2000 PMC97386

[33] Johnson M, Cockayne A, Morrissey JA (2008) Iron-regulated biofilm formation in *Staphylococcus aureus* Newman requires ica and the secreted protein Emp. Infect Immun 76: 1756–65. 18268030 10.1128/IAI.01635-07 PMC2292859

[34] Luong TT, Lee CY (2007) Improved single-copy integration vectors for *Staphylococcus aureus.* . J Microbiol Methods 70: 186–90. 17512993 10.1016/j.mimet.2007.04.007 PMC2001203

[35] Collins J, Buckling A, Massey RC (2008) Identification of Factors Contributing to T-Cell Toxicity of *Staphylococcus aureus* Clinical Isolates. J Clin Microbiol 46: 2112–4. 18417658 10.1128/JCM.00156-08 PMC2446864

[36] Wang JE, Jørgensen PF, Almlöf M, Thiemermann C, Foster SJ, et al (2000) Peptidoglycan and lipoteichoic acid from *Staphylococcus aureus* induce tumor necrosis factor alpha, interleukin 6 (IL-6), and IL-10 production in both T cells and monocytes in a human whole blood model. Infect Immun 68: 3965–70. 10858210 10.1128/iai.68.7.3965-3970.2000 PMC101674

[37] Wang JE, Jørgensen PF, Almlöf M, Thiemermann C, Foster SJ, et al (2000) Peptidoglycan and lipoteichoic acid from *Staphylococcus aureus* induce tumor necrosis factor alpha, interleukin 6 (IL-6), and IL-10 production in both T cells and monocytes in a human whole blood model. Infect Immun 68: 3965–3970. 10858210 10.1128/iai.68.7.3965-3970.2000 PMC101674

[38] Spentzas T, Kudumula R, Acuna C, Talati AJ, Ingram KC, et al (2011) Role of bacterial components in macrophage activation by the LAC and MW2 strains of community-associated, methicillin-resistant *Staphylococcus aureus* . Cell Immunol 269: 46–53. 21458780 10.1016/j.cellimm.2011.03.009

[39] Madge LA, Pober JS (2001) TNF signaling in vascular endothelial cells. Exp Mol Pathol. 70: 317–25. 10.1006/exmp.2001.2368 11418010

[40] Cheung AL, Koomey JM, Lee S, Jaffe EA, Fischetti VA (1991) Recombinant human tumor necrosis factor alpha promotes adherence of *Staphylococcus aureus* to cultured human endothelial cells. Infect Immun 59: 3827–31. 1894381 10.1128/iai.59.10.3827-3831.1991 PMC258959

[41] Hartleib J, Köhler N, Dickinson RB, Chhatwal GS, Sixma JJ, et al (2000) Protein A is the von Willebrand factor binding protein on *Staphylococcus aureus*. Blood. 96: 2149–56. 10979960

[42] Peerschke EI, Bayer AS, Ghebrehiwet B, Xiong YQ (2006) gC1qR/p33 blockade reduces *Staphylococcus aureus* colonization of target tissues in an animal model of infective endocarditis. Infect Immun. 74: 4418–23. 10.1128/IAI.01794-05 PMC1539591 16861627

[43] Sethi S, Herrmann M, Roller J, von Müller L, Peerschke EI, et al (2011) Blockade of gC1qR/p33, a receptor for C1q, inhibits adherence of *Staphylococcus aureus* to the microvascular endothelium. Microvasc Res. 82: 66–72. 10.1016/j.mvr.2011.04.007 PMC3114305 21539847

[44] Oviedo-Boyso J, Barriga-Rivera JG, Valdez-Alarcón JJ, Bravo-Patiño A, Cárabez-Trejo A, et al (2008) Internalization of *Staphylococcus aureus* by bovine endothelial cells is associated with the activity state of NF-kappaB and modulated by the pro-inflammatory cytokines TNF-alpha and IL-1beta. Scand J Immunol. 67: 169–76. 10.1111/j.1365-3083.2007.02056.x 18201371

[45] Oviedo-Boyso J, Cardoso-Correa BI, Cajero-Juárez M, Bravo-Patiño A, Valdez-Alarcón JJ, et al (2008) The capacity of bovine endothelial cells to eliminate intracellular *Staphylococcus aureus* and *Staphylococcus epidermidis* is increased by the proinflammatory cytokines TNF-alpha and IL-1beta. FEMS Immunol Med Microbiol. 54: 53–9. 10.1111/j.1574-695X.2008.00447.x 18625014

[46] Braun L, Ghebrehiwet B, Cossart P (2000) gC1q-R/p32, a C1q-binding protein, is a receptor for the InlB invasion protein of Listeria monocytogenes. EMBO J 19: 1458–1466. 10747014 10.1093/emboj/19.7.1458 PMC310215

[47] Novick R (1967) Properties of a cryptic high-frequency transducing phage in *Staphylococcus aureus* . Virology 33: 155–66. 4227577 10.1016/0042-6822(67)90105-5

[48] Novick RP (1991) Genetic systems in staphylococci. Methods Enzymol. 204: 587–636. 10.1016/0076-6879(91)04029-n 1658572

[49] Greene C, McDevitt D, Francois P, Vaudaux PE, Lew DP, et al (1995) Adhesion properties of mutants of *Staphylococcus aureus* defective in fibronectin-binding proteins and studies on the expression of *fnb* genes. Mol Microbiol 17: 1143–1152. 8594333 10.1111/j.1365-2958.1995.mmi_17061143.x

[50] Kreiswirth BN, Löfdahl S, Betley MJ, O’Reilly M, Schlievert PM, et al (1983) The toxic shock syndrome exotoxin structural gene is not detectably transmitted by a prophage. Nature 305: 709–712. 6226876 10.1038/305709a0

[51] Duthie ES, Lorenz LL (1952) Staphylococcal coagulase: mode of action and antigenicity. J Gen Microbiol 6: 95–107. 14927856 10.1099/00221287-6-1-2-95

[52] McDevitt D, Francois P, Vaudaux P, Foster TJ (1995) Identification of the ligand-binding domain of the surface-located fibrinogen receptor (clumping factor) of *Staphylococcus aureus* . Mol Microbiol 16: 895–907. 7476187 10.1111/j.1365-2958.1995.tb02316.x

[53] McDevitt D, Francois P, Vaudaux P, Foster TJ (1994) Molecular characterization of the clumping factor (fibrinogen receptor) of *Staphylococcus aureus*. Mol Microbiol. 11: 237–48. 10.1111/j.1365-2958.1994.tb00304.x 8170386

[54] McDevitt D, Vaudaux P, Foster TJ (1992) Genetic evidence that bound coagulase of *Staphylococcus aureus* is not clumping factor. Infect Immun 60: 1514–1523. 1548075 10.1128/iai.60.4.1514-1523.1992 PMC257025

[55] Sau S, Bhasin N, Wann ER, Lee JC, Foster TJ, et al (1997) The *Staphylococcus aureus* allelic genetic loci for serotype 5 and 8 capsule expression contain the type-specific genes flanked by common genes. Microbiology 143: 2395–2405. 9245821 10.1099/00221287-143-7-2395

